# Compliance and persistence with oral bisphosphonates for the treatment of osteoporosis in female patients with rheumatoid arthritis

**DOI:** 10.1186/s12891-017-1514-4

**Published:** 2017-04-11

**Authors:** Ji-Heh Park, Eun-Kyoung Park, Dong-Wan Koo, Shinwon Lee, Sun-Hee Lee, Geun-Tae Kim, Seung-Geun Lee

**Affiliations:** 1 Division of Rheumatology, Department of Internal Medicine, Pusan National University School of Medicine, Pusan National University Hospital, 179 Gudeok-Ro, Seo-Gu, 49241 Busan, South Korea; 2 Department of Internal Medicine, Dong-Eui Hospital, Dong-Eui University College of Oriental Medicine, Busan, South Korea; 3 Department of Internal Medicine, Pusan National University School of Medicine, Medical Research Institute, Pusan National University Hospital, Busan, South Korea; 4 Division of Rheumatology, Department of Internal Medicine, Kosin University College of Medicine, Busan, South Korea

**Keywords:** Rheumatoid arthritis, Osteoporosis, Diphosphonates, Compliance, Medication adherence

## Abstract

**Background:**

Poor adherence with oral bisphosphonates (BPs) can mitigate their therapeutic benefit for osteoporosis and is a significant clinical burden. Most previous studies regarding adherence with oral BPs have focused on postmenopausal osteoporosis, but little attention has been given to patients with rheumatoid arthritis (RA). Thus, we investigated compliance and persistence with oral BPs in the treatment of osteoporosis and analyzed risk factors for poor adherence in female patients with (RA) in real setting.

**Methods:**

This is a retrospective longitudinal study including 396 female patients with RA in whom oral BPs were newly initiated from Aug 2004 to Aug 2014 at a university rheumatology center in South Korea. Compliance was quantified using the 1-year medication possession ratio (MPR), while persistence was defined as duration from the initiation to the end of BPs therapy without interruption exceeding 56 days. Seropositve RA was defined as having a positive test result for the presence of either rheumatoid factor or anti-cyclic citrullinated peptide antibody.

**Results:**

Of 396 RA patients, 221 (55.8%) were prescribed risedronate 35 mg weekly; 17 (4.3%) received alendronate 70 mg weekly; and 158 (39.9%) received ibandronate 150 mg monthly. The 1-year MPR was 70.1% and the proportion of RA patients with the 1-year MPR ≥ 0.8 was 60.1%. A total of 274 (69.2%) patients discontinued oral BPs during the study period and persistence with BPs was 63.3% at 1 year, 50.7% at 2 years and 33.3% at 3 years. The most common cause of non-persistence was adverse events (47.5%), followed by poor health literacy (40.5%) and cost (12%). Both compliance and persistence with monthly oral BPs were significantly lower than those with weekly regimens (OR: 2.48, 95% CI: 1.59–3.89, *P* < 0.001 and HR: 2.19, 95% CI: 1.69–2.83, *P* < 0.001, respectively). Additionally, patients with seropositive RA showed better compliance and persistence with BPs compared with their seronegative counterparts.

**Conclusions:**

Compliance and persistence with oral BPs in RA patients were suboptimal in real practice, thereby limiting the efficacy of osteoporosis treatment. Extending the dosing interval of BPs may improve medication adherence in RA patients.

## Background

Rheumatoid arthritis (RA) is a chronic inflammatory arthritis with unknown etiology characterized by progressive and systemic inflammation, resulting in joint destruction and functional disability. Due to inflammation-induced osteoclast activation, the effect of glucocorticoids and other factors including increased disease activity, decreased physical function and lower body mass index (BMI), RA has been identified as a critical risk factor for osteoporosis [1, 2], and subsequently predisposes patients to increased risk of vertebral and non-vertebral fracture [3]. Since osteoporotic fracture can cause progressive pain, impair quality of life, and induce significant morbidity and mortality as well as increased healthcare costs [4], the appropriate screening for systemic bone loss and treatment of osteoporosis to minimize fracture risk in patients with RA is of paramount importance. However, the overall management of osteoporosis in patients with RA is inadequate in real practice [5, 6], which has been recognized as a major concern in rheumatology.

Bisphosphonates (BPs), a class of drugs that inhibit osteoclast-mediated bone resorption, is the first-line treatment option for osteoporosis. Their efficacy in increasing bone mineral density (BMD) and normalizing bone turnover is well established in postmenopausal osteoporosis (PMO) and glucocorticoid-induced osteoporosis (GIOP) [7] and approximately 50% of osteoporotic fractures can be prevented through the use of BPs [8]. A recent meta-analysis also demonstrated that BPs can preserve BMD and prevent vertebral fractures in patients with rheumatic diseases including RA [9], however, due to lack of patient education regarding the risks related to osteoporosis, gastrointestinal adverse effects and the complexity of the recommended regimen, adherence with oral BPs has been reported to be suboptimal in clinical practice [10–12], which consequently increases the risk of osteoporotic fractures by 30–40% [13]. Thus, poor adherence with oral BPs can mitigate their therapeutic benefit and is a significant clinical burden [14].

Adherence is a general term encompassing both compliance and persistence [15]. Compliance is defined as the extent to which a patient follows the prescribed interval and dose of treatment regimens whereas persistence is defined as the cumulative time duration from initiation to discontinuation of therapy [15]. Most previous studies regarding adherence with oral BPs have focused on PMO [10, 11] but little attention has been given to patients with RA. Considering a significant association between RA and risk of osteoporosis, characterizing the adherence of oral BPs in RA patients and understanding associated factors for poor adherence are crucial to improving clinical outcome. Thus, in the present study, we investigated compliance and persistence with oral BPs in the treatment of osteoporosis in female patients with RA and analyzed risk factors for poor adherence in the real clinical setting.

## Methods

### Study design and subjects

In this retrospective longitudinal study, we recruited 396 female patients with RA aged 20 years or older in whom oral BPs were newly initiated for the treatment of osteoporosis from Aug 2004 to Aug 2014 at a university-affiliated rheumatology center in South Korea. All patients fulfilled the American College of Rheumatology 1987 revised classification criteria for RA [16]. The following oral BPs available in our center during the study period were investigated: risedronate 35 mg weekly; alendronate 70 mg weekly; and ibandronate 150 mg monthly. All prescriptions of oral BPs were made by experienced rheumatologists. The index date was defined as the date of initial prescription of oral BPs and study subjects were followed up until Dec 2015 to assess treatment adherence. To select new oral BP users, RA patients who had received any prescription related to osteoporosis such as BPs, selective estrogen receptor modulators or teriparatide during 12 months before the index date were excluded; however, patients who consumed calcium or vitamin D supplements were included. To ensure correct identification of BP initiation and sufficient data for estimation of adherence, all patients were required to have medical record for 12 months preceding and following their index date [17]. Subjects having bone metastasis or Paget’s disease requiring BPs were excluded. The study protocol was approved by the Research and Ethical Review Board of the Pusan National University Hospital, which waived informed consent.

### Measurement of compliance and persistence

Compliance was quantified by the medication possession ratio (MPR), which is defined as the proportion of duration during which the medication has been actually dispensed over a fixed observation period. In this study, the 1-year MPR was calculated as the percentage of number of available BPs supply days during a predefined period of 365 days for each RA patient. Subjects were considered to be compliant with oral BPs if the 1-year MPR was ≥ 0.8 and non-compliant if the 1-year MPR was < 0.8, as previously indicated [18–23]. Persistence was defined as the number of days on oral BPs treatment from the index date to the end of duration of the last prescription or the end of study period (31 Dec 2015) without interruption (permissible gap) exceeding 56 days [10, 24]. To avoid underestimating true persistence, the treatment was considered to be persistent if the oral BP was switched to another oral BPs or the dose of drugs was changed within a permissible gap [19, 24, 25]. Study subjects who discontinued BPs for a duration longer than permissible gap were considered to be non-persistent, even if BPs were subsequently restarted [20]. Reasons for non-persistence with oral BPs were identified and classified as adverse events, poor health literacy and cost. Adverse events were further categorized as gastrointestinal complaints, osteonecrosis of jaw (ONJ), myalgia/arthralgia, kidney injury and cardioplumonary disorders based on patients’ report or the decision of investigator. The category “poor health literacy” indicates discontinuation of oral BPs due to unawareness of the significance and requirements of regular drug use and withdrawal of oral BPs due to economic problems was classified as “cost”. “Poor health literacy” and “cost” were determined according to patients’ report from their medical chart reviewing.

### Clinical assessment

The following demographics and clinical parameters of all patients with RA were collected by reviewing medical records: age, disease duration, concurrent mediations, previous history of fracture, baseline dual energy X-ray absorptiometry (DEXA) and BMD, reimbursement of BPs, BMI, comorbidities including hypertension (HTN) and type 2 diabetes mellitus (DM), erythrocyte sedimentation rate (ESR), immunoglobulin M-rheumatoid factor (RF), anti-cyclic citrullinated peptide (CCP) antibody and disease activity score assessed using the 28-joint count for swelling and tenderness (DAS28)-ESR. Age was assessed at the index date and BMI was calculated by dividing body weight by the square of height in meters (kg/m^2^). Disease duration was dichotomized as <24 months or ≥24 months because “early RA” is traditionally defined as a disease duration of less than 24 months according to previous studies [26, 27]. Purchasing National Health Insurance Service (NHIS) is mandatory for all citizens in South Korea and RA patients can receive reimbursement for BPs if the T-score in the L14 spine, femoral neck or total hip is less than −2.5, which is determined by the Ministry of Health and Welfare. Accordingly, we recorded the presence of reimbursement for BPs by NHIS in all patients. Comorbidities were defined as morbidities developed before the index date and HTN was defined as blood pressure ≥ 140/90 mmHg or requiring antihypertensive drugs. Type 2 DM was defined according to the World Health Organization criteria or requiring hypoglycemic medications. RF was measured using a particle-enhanced immunoturbidimetric assay (range 0–14 IU/ml) and anti-CCP was assessed by a chemiluminescent microparticle immunoassay (range 0–5 U/mL). Seropositve RA was defined as having a positive test result for the presence of either RF or anti-CCP antibody, whereas a patient was categorized as seronegative RA if both RF and anti-CCP antibody were negative. DAS28-ESR score was calculated by the following formula: DAS28 ‐ ESR score = [0.56 × √ (TJC 28)] + [0.28 × √ (SJC 28)] + [0.70 × ln ESR] + [0.0014 × visual analog scale score ] [28].

### Statistical analyses

Continuous variables are summarized as mean ± standard deviation or median (interquartile range) and categorical variables as the number of cases with percentages. The Kolmogorov-Smirnov test was performed to assess the distribution of continuous variables. For group comparisons, Student’s *t* test or the Mann–Whitney *U* test was used for continuous variables and the *χ* ^2^ test or Fisher’s exact test was conducted for categorical variables, as appropriate. Multivariable backward logistic regression models including variables with *P* <0.2 in the univariable analyses were used to identify risk factors for being non-compliant (MPR < 0.8) to oral BPs. Persistence was analyzed and plotted using Kaplan-Meier method and compared using the log-rank test. Multivariable Cox proportional hazard regression models with backward selection were employed to determine predictors for non-persistence to oral BPs over the follow-up period. Covariates with *P* <0.2 in univariable Cox regression analyses were incorporated into multivariable models. Age and the presence of reimbursement of BPs were considered as a priori confounders and included in both multivariable logistic and Cox regression models. Statistical significance was defined as *P* <0.05. All analyses were conducted using STATA 11.0 for Windows (StataCorp LP, College Station, TX, USA) and PASW18.0 for Windows (Chicago, IL, USA).

## Results

Baseline clinical characteristics of total study subjects are shown in Table 1. The mean age was 65.9 ± 11.3 years and 324 (81.8%) patients were classified as having seropositive RA. Baseline DEXA was performed in 355 (89.6%) patients and 29 (7.3%) subjects had previous fracture history. More than half of the patients with RA (64.1%) received reimbursement for BPs by the NHIS. Of 396 patients with RA, 221 (55.8%) were prescribed with risedronate 35 mg weekly; 17 (4.3%) received alendronate 70 mg weekly; and 158 (39.9%) received ibandronate 150 mg monthly. Thus, patients were grouped according to the dosing frequency of treatment they received: weekly oral BPs, *n* = 238 (60.1%); and monthly oral BPs, *n* = 158 (39.9%). The frequency of baseline DEXA in RA patients treated with monthly BPs was significantly higher than in those received weekly BPs (Table 1). However, other demographic and clinical characteristics were comparable between study subjects treated with monthly and weekly oral BPs.

**Table 1:** Clinical characteristics of female patients with rheumatoid arthritis receiving oral bisphosphonates

	Monthly BP (*n* = 158)	Weekly BP (*n* = 238)	*p* value	Total subjects (*n* = 396)
Age, years, mean ± SD	66.5 ± 10.5	65.6 ± 11.9	0.416	65.9 ± 11.3
Disease duration, months, median (IQR)	42 (12.5–82)	35 (18–86)	0.7	39 (18–84)
Seropositive RA, n (%)	131 (82.9)	193 (81.1)	0.646	324 (81.8)
DAS28-ESR, mean ± SD	3.53 ± 1.67	3.84 ± 1.67	0.089	3.72 ± 1.68
Concurrent medications				
Methotrexate, n (%)	93 (58.9)	149 (62.6)	0.454	242 (61.1)
Sulfasalazine, n (%)	18 (11.4)	37 (15.5)	0.242	55 (13.9)
Hydroxychloroquine, n (%)	86 (54.4)	121 (50.8)	0.484	207 (52.3)
Leflunomide, n (%)	29 (18.4)	49 (20.6)	0.584	79 (19.7)
Glucocorticoid, n (%)	138 (87.9)	206 (86.6)	0.697	344 (86.9)
Glucocorticoid dose, mg, median (IQR)	7.5 (5–10)	7.5 (5–10)	0.631	7.5 (5–10)
Calcium/vitamin D, n (%)	32 (20.3)	42 (17.6)	0.514	74 (18.7)
Previous history of fracture, n (%)	11 (7)	18 (7.6)	0.822	29 (7.3)
Baseline DEXA, n (%)	152 (96.2)	203 (85.3)	<0.001	355 (89.6)
BMD (T score)				
Spine, mean ± SD	−1.8 ± 1.4	−1.7 ± 1.4	0.629	−1.7 ± 1.4
Femoral neck, mean ± SD	−1.5 ± 1.1	−1.4 ± 1.1	0.273	−1.5 ± 1.1
Total hip, mean ± SD	− 1.3 ± 1.1	− 1.3 ± 1.1	0.595	−1.3 ± 1.1
Reimbursement for BPs	108 (68.4%)	146 (61.3%)	0.154	254 (64.1%)
BMI, mean ± SD	22.4 ± 2.8	22.2 ± 3.2	0.435	22.3 ± 3
Comorbidity				
Type 2 DM, n (%)	19 (12)	16 (6.7)	0.069	35 (8.8)
HTN, n (%)	42 (26.6)	56 (23.5)	0.342	98 (24.7)

*RA* rheumatoid arthritis, *DEXA* dual energy X-ray absorptiometry, *BMD* bone mineral density, *BPs* bisphosphonates, *BMI* body mass index, *DM* diabetes mellitus, *HTN* hypertension

The 1-year MPR in all patients was 71% (95% CI: 67.2–74.8) and the proportion of subjects compliant with BPs (the 1-year MPR ≥ 0.8) was 60.1%. RA patients received monthly BPs showed a significantly better 1-year MPR than did those received weekly BPs (80.2% vs. 64.9%, *P* < 0.001). In addition, the mean 1-year MPR in RA patients with seropositive RA, disease duration ≥24 months or not taking calcium/vitamin D supplement was significantly higher than in RA patients without these features (74.9% vs. 53.2%, *P* < 0.001, 75.5% vs 62.6%, *P* = 0.002, and 73.8% vs 58.7%, *P* = 0.005, respectively). Table 2 summarizes risk factors for non-compliance with oral BPs which was defined as the 1-year MPR < 0.8. In univariable logistic regression analyses, weekly BPs and calcium/vitamin D supplement were significantly related with subjects who were non-complaint with BPs, while seropositive RA and disease duration ≥ 24 months showed a significant association with better compliance (Table 2). After adjusting for confounding factors, weekly BPs (OR: 2.48, 95% CI: 1.59–3.89, *P* < 0.001) and calcium/vitamin D supplement (OR: 1.8, 95% CI: 1.04–3.12, *P* = 0.037) were independent risk factors for non-compliance with BPs. Otherwise, patients with seropositive RA showed a significantly lower probability of being non-complaint with BPs in multivariable logistic model (OR: 0.51, 95% CI: 0.29–0.89, *P* = 0.019).

**Table 2:** Risk factors for ^a^non-compliance to bisphosphonates in patients with rheumatoid arthritis

Variables	Univariable		Multivariable	
	OR (95% CI)	*p* value	^b^OR (95% CI)	*p* value
Dosing frequency weekly vs. monthly BPs	2.42 (1.57–3.73)	<0.001	2.48 (1.59–3.89)	<0.001
Seropositive RA	0.43 (0.26–0.73)	0.002	0.51 (0.29–0.89)	0.019
Disease duration ≥ 24 months	0.48 (0.31–0.73)	0.001	0.66 (0.41–1.05)	0.077
Calcium /vitamin D supplement	2.01 (1.21–3.34)	0.007	1.8 (1.04–3.12)	0.037
Previous compression fracture	0.9 (0.42–1.97)	0.8		
Reimbursement for BPs	1.15 (0.75–1.15)	0.519	-	-
Age, years	1 (0.99–1.02)	0.632	-	-
Glucocorticoids use	0.79 (0.44–1.42)	0.427		
Baseline DEXA	0.68 (0.35–1.29)	0.236		
DAS28-ESR	1.05 (0.92–1.19)	0.477		

*BPs* bisphosphonates, *RA* rheumatoid arthritis, *DEXA* dual energy X-ray absorptiometry, *DAS28-ESR* disease activity score assessed using the 28-joint count for swelling and tenderness- erythrocyte sedimentation rate

^a^Non-compliance was defined if the 1-year medication possession ratio was less than 0.8

^b^Estimated using multivariable backward logistic regression models including dosing frequency of BPs, seropositive RA, disease duration > 24 months, calcium /vitamin D supplement, age and reimbursement for BPs

**Fig. 1:**
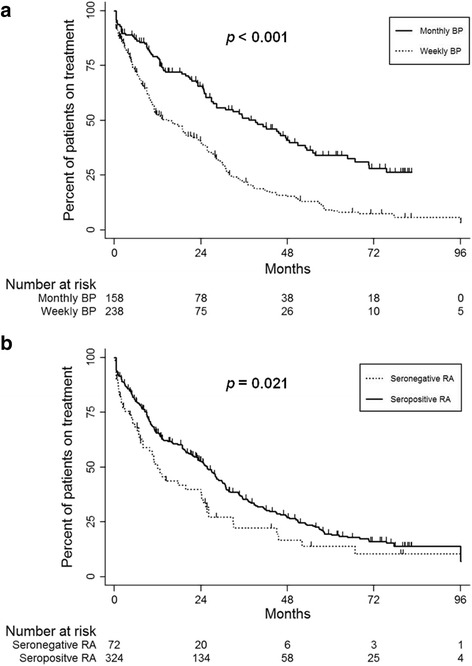
Persistence with oral bisphosphonates treatment by dosing frequency (**a**) and seropositive rheumatoid arthritis (**b**)

A total of 274 (69.2%) patients discontinued oral BPs during the study period. The reasons for BP withdrawal were as follows: adverse events (47.5%), poor health literacy (40.5%) and cost (12%), as shown in Table 3. The most common cause of adverse events was gastrointestinal complaints (40.9%) and 4 (1.5%) patients stopped BPs due to ONJ. The median time until patients discontinued BPs was 24.2 months (95% CI: 20.4–28.1) and persistence with BPs was 63.3% (95% CI: 58.1–68) at 1 year, 50.7% (95% CI: 45.3–55.8) at 2 years and 33.3% (95% CI: 28.1–30.3) at 3 years, as estimated by Kaplan-Meier method. As illustrated in Fig. 1, the monthly regimen of BPs showed significantly a longer persistence than did weekly BPs (*P* < 0.001), whereas persistence with oral BPs in patients with seropositive RA was significantly higher than in those with seronegative RA (*P* = 0.021).

**Table 3:** Reasons for non-persistence to bisphosphonates in patients with rheumatoid arthritis

	Non-persistence to BPs (*n* = 274)
Adverse events, n (%)	130 (47.5)
Gastrointestinal complaint, n (%)	112 (40.9)
Myalgia/arthralgia, n (%)	9 (3.3)
ONJ, n (%)	4 (1.5)
Kidney injury, n (%)	3 (1.1)
Cardioplumonary disorder, n (%)	2 (0.7)
Poor healthy literacy, n (%)	111 (40.5)
Cost, n (%)	33 (12)

*ONJ* Osteonecrosis of jaw

Univariable Cox regression analyses revealed that dosing frequency of BPs, seropositive RA and disease duration were significantly related with non-persistence with BPs (Table 4). RA patients with previous compression fracture showed a higher risk for discontinuation of BPs, but it did not reach statistical significance. In multivariable Cox regression models, weekly regimen was a significant determinant for non-persistence with BPs (HR: 2.19, 95% CI: 1.69–2.83, *P* < 0.001). Patients with seropositive RA persisted with BPs treatment significantly longer than those with seronegative RA after adjusting for confounding factors (HR: 0.68, 95% CI: 0.49–0.92, *P* = 0.014). In the cause-specific analyses for drug persistence, weekly BPs showed worse persistence due to adverse events (HR: 5.41, 95% CI: 3.34–8.76, *P* < 0.001) and poor health literacy (HR: 1.49, 95% CI: 0.96–2.31, *P* = 0.077) than monthly BPs in multivariable Cox models, but this association was not observed for non-persistence due to cost (data not shown).

**Table 4:** Predictors for non-persistence to bisphosphonates in patients with rheumatoid arthritis

Variables	Univariable		^a^Multivariable	
	HR (95% CI)	*p* value	HR (95% CI)	*p* value
Dosing frequency weekly vs. monthly BPs	2.16 (1.67–2.79)	<0.001	2.19 (1.69–2.83)	<0.001
Seropositive RA	0.69 (0.51–0.95)	0.023	0.68 (0.49–0.92)	0.014
Disease duration ≥ 24 months	0.69 (0.54–0.88)	0.003	-	-
Previous compression fracture	1.44 (0.98–2.12)	0.062	-	-
Reimbursement for BPs	0.85 (0.66–1.08)	0.192	-	-
Age, years	0.99 (0.98–1.01)	0.604	-	-
Glucocorticoids use	1.06 (0.72–1.55)	0.763		
Calcium /vitamin D supplement	1.08 (0.78–1.5)	0.622		
Baseline DEXA	0.84 (0.57–1.24)	0.373		
DAS28-ESR	1.02 (0.95–1.09)	0.586		

*BPs* Bisphosphonates, *RA* Rheumatoid arthritis, *DEXA* dual energy X-ray absorptiometry, *DAS28-ESR* disease activity score assessed using the 28-joint count for swelling and tenderness- erythrocyte sedimentation rate

^a^Estimated using multivariable backward logistic regression models including dosing frequency of BPs, seropositive RA, disease duration > 24 months, previous compression fracture, reimbursement for BPs and age

## Discussion

Our retrospective longitudinal study revealed that the overall adherence with oral BPs in patients with RA was suboptimal in the routine practice setting. The 1-year MPR was 70.1% and only 63.3% of patients with RA were still persistent with BPs treatment after 1 year. The persistence rate gradually decreased over the study period, but the highest discontinuation rate was seen within the first year after BP initiation. The most common cause of non-persistence with BPs was adverse events, followed by poor health literacy and cost. Both compliance and persistence with monthly oral BPs were significantly higher than those with weekly regimens, suggesting that dosing frequency may be a contributing factor for adherence with BPs in RA patients. Of interest, patients with seropositive RA showed better adherence with BPs in comparison with their seronegative counterparts.

While previous researches have consistently demonstrated insufficient adherence with BPs in PMO patients with the 1-year MPR and persistence rate ranging between 58 and 81% and 24 and 69%, respectively [10, 11], little is known about compliance and persistence with BPs in RA patients. To our knowledge, this is one of the first studies investigating adherence of osteoporosis medications primarily focusing on female patients with RA. Several previous studies concerning adherence of BPs in PMO included RA patients, but subgroup analysis regarding RA was not performed and RA-related data were limited [21, 22, 29]. In addition, Richards et al. reported a poor adherence with BPs in US veterans with RA using Pharmacy Benefit Management data [18], but more than 90% of subjects were male in their study. Given that the majority of RA patients are female, findings by Richards et al. may not be generalizable to the entire RA population. Taken together, our study can provide more comprehensive data regarding adherence with BPs in patients with RA than do previous studies.

Although Cotte et al. reported that adherence of osteoporosis medication in RA patients was lower than that in non-RA subjects using medical claim data [29], the 1-year MPR and persistence (70.1% and 63.3%, respectively) in patients with RA in our data seemed to be comparable to those in PMO reported in meta-analyses [10, 11]. Given that comorbidity is generally recognized as a risk factor for poor adherence with osteoporosis treatment [30], the worse compliance and persistence in RA patients in our study may be underestimated. Whether study subjects truly consumed their prescribed oral BPs could not be evaluated in our study, which may have contributed to this underestimation. However, since various factors including the methodology of study, race and healthcare system can affect adherence with BPs, direct comparisons of adherence between different studies should be made cautiously. Thus, it is beyond the scope of this study to demonstrate whether RA patients showed worse adherence than PMO subjects and further researches are needed.

One of the major findings in this study was that adherence with BPs in patients with RA was suboptimal in real practice. Recent evidence has been indicated that good adherence with BP could substantially improve clinical outcomes such as fracture prevention in PMO [13]. Although the association between adherence with anti-resorptives and fracture risk in RA has not yet been determined, it seems plausible that these results regarding PMO are extrapolated to RA since the mechanisms of BPs probably do not differ between PMO and RA [31]. In particular, Feng et al. recently noticed that the effect of BPs on osteoporosis treatment occurred after 36 months in patient with rheumatic diseases [9], thereby highlighting the importance of long-term persistence. Thus, it is assumed that inadequate adherence with BPs can also negatively impact clinical outcome of osteoporosis in RA. Considering our results, it is necessary to improve compliance and persistence of osteoporotic treatments in RA patients in clinical practice.

Adverse events were one of the main reasons of non-persistence with BPs and gastrointestinal complaint was the most common cause among adverse events in RA patients in this study, which agrees with previous reports investigating BPs persistence in PMO subjects [32, 33]. As ONJ and atypical femoral fractures are associated with BPs and have attracted growing public attention in South Korea, the fear of these adverse drug reactions among patients may have had negative effects on adherence to the use of BPs in our study. Of note, our data shows that approximately 40% of RA patients stopped oral BPs due to unawareness of the significance of osteoporosis treatment (poor health literacy). Since osteoporosis has an asymptomatic nature, patients may be skeptical of the efficacy of treatment and not fully appreciate the complex dosing requirements of oral BPs interfering with daily life [34], which may contribute to non-persistence due to poor health literacy in our study. In addition, this finding warrants further studies investigating the effect of patient education specifically targeting osteoporosis on adherence with anti-resorptive therapy in RA subjects.

Determinants of adherence with BPs in patients with RA were analyzed in our study. First, as expected, the monthly regimen independently correlated with better compliance and persistence compared with weekly BPs. Previous researches also reported that a less-frequent dosing interval was significantly related with better adherence with BPs in PMO [35, 36], which is attributable to the convenience of a dosing schedule. Thus, we expected that the extending dosing interval of oral BPs may improve treatment adherence in RA patients. Second, patients with seropositive RA showed better adherence with BPs than those with seronegative RA. Although the reason for this is unclear, we speculate that worse disease course in seropositive RA patients compared with their seronegative counterparts would contribute to heightened awareness of health status including osteoporosis, thereby subsequently resulting in a better adherence with BPs. Third, contrary to the initial expectation, the presence of reimbursement of BPs was not associated with drug adherence. In South Korea, the average monthly cost of BPs was approximately $20 (US dollars) if no reimbursement is given. Relatively low cost BPs might enhance overall availability of medications and not significantly affect the adherence of treatment. Last, calcium and vitamin D supplementation, possibly considered as an indicator of higher motivation, was reported to be associated with better compliance with BPs in PMO patients [29], but reverse association was observed in patients with RA in our data. We speculate that our RA subjects may prefer calcium and vitamin D supplementation to oral BPs due to cost and convenience, thus such dietary intake might replace BPs during study period, but the causal relationship is uncertain. Further investigations are needed to determine the impact of calcium and vitamin D supplementation on adherence of BPs treatment in RA.

To analyze medication persistence, a prespecified limit on the number of days allowed between refills, which is termed as the “permissible gap,” must be included [15] and non-persistence was assumed if the period for drug discontinuation was longer than this gap [10, 24, 30]. However, there is no uniform definition of permissible gap in the literature and this gap ranged from 15 to 90 days, according to several previous studies [10]. Because the methods for determining the permissible gap should be based on the pharmacologic properties of medication, the treatment regime, and the characteristics of the subjects receiving drugs [15], it is difficult to establish the ideal value of a permissible gap.

This study has potential limitations that are inherent to its retrospective observational design in a single center. First, the superior adherence of patients prescribed monthly BPs over the weekly regimen need to be interpreted carefully since baseline clinical characteristics between dosing frequency of BPs were not randomized. However, except for the frequency of DEXA, no significant differences in baseline features according to the dosing regimen were observed. Second, because only RA patients visited a tertiary university hospital were included in our study, there may be selection bias. Third, information about the communication between RA patients and healthcare professionals were not assessed. As shown by Kishmoto et al. [35], providing a more detailed explanation regarding the disease and health status to patients by their rheumatologists may improve treatment adherence in osteoporosis. Last, potential confounding factors such as household income, educational level, urban–rural dwelling status and comorbidities other than HTN and type 2 DM were not fully assessed.

## Conclusions

To summary, the compliance and persistence with oral BPs in female patients with RA was suboptimal in real practice, thereby potentially limiting the efficacy of treatment for osteoporosis. Adverse events due to gastrointestinal complaint and lack of patient awareness of the significance and requirements of BPs therapy were the major causes of non-persistence, which suggest that appropriate patient education is needed to increase knowledge of osteoporosis to improve treatment adherence in RA subjects. In term of treatment adherence, monthly BPs produced better compliance and persistence than did weekly BPs in RA subject, thus less frequent administration of BPs may contribute to enhanced medication adherence and subsequently optimize clinical outcomes. However, further work is needed to confirm our findings.

## References

[1] Lee SG, Park YE, Park SH, Kim TK, Choi HJ, Lee SJ, Kim SI, Lee SH, Kim GT, Lee JW (2012). Increased frequency of osteoporosis and BMD below the expected range for age among South Korean women with rheumatoid arthritis. Int J Rheum Dis.

[2] Lee JH, Sung YK, Choi CB, Cho SK, Bang SY, Choe JY, Hong SJ, Jun JB, Kim TH, Lee J (2016). The frequency of and risk factors for osteoporosis in Korean patients with rheumatoid arthritis. BMC Musculoskeletal Disord.

[3] Kim SY, Schneeweiss S, Liu J, Daniel GW, Chang CL, Garneau K, Solomon DH (2010). Risk of osteoporotic fracture in a large population-based cohort of patients with rheumatoid arthritis. Arthritis Res Ther.

[4] Papaioannou A, Kennedy CC, Ioannidis G, Sawka A, Hopman WM, Pickard L, Brown JP, Josse RG, Kaiser S, Anastassiades T (2009). The impact of incident fractures on health-related quality of life: 5 years of data from the Canadian Multicentre Osteoporosis Study. Osteoporos Int.

[5] Solomon DH, Katz JN, Jacobs JP, La Tourette AM, Coblyn J (2002). Management of glucocorticoid-induced osteoporosis in patients with rheumatoid arthritis: rates and predictors of care in an academic rheumatology practice. Arthritis Rheum.

[6] Lee JH, Cho SK, Han M, Kim D, Bae SC, Sung YK (2014). Are glucocorticoid-induced osteoporosis recommendations sufficient to determine antiosteoporotic treatment for patients with rheumatoid arthritis?. Korean J Intern Med.

[7] Mullen MB, Saag KG (2015). Evaluating and mitigating fracture risk in established rheumatoid arthritis. Best Pract Res Clin Rheumatol.

[8] Geusens PP, Roux CH, Reid DM, Lems WF, Adami S, Adachi JD, Sambrook PN, Saag KG, Lane NE, Hochberg MC (2008). Drug Insight: choosing a drug treatment strategy for women with osteoporosis-an evidence--based clinical perspective. Nat Clin Pract Rheumatol.

[9] Feng Z, Zeng S, Wang Y, Zheng Z, Chen Z (2013). Bisphosphonates for the prevention and treatment of osteoporosis in patients with rheumatic diseases: a systematic review and meta-analysis. PLoS One.

[10] Karlsson L, Lundkvist J, Psachoulia E, Intorcia M, Strom O (2015). Persistence with denosumab and persistence with oral bisphosphonates for the treatment of postmenopausal osteoporosis: a retrospective, observational study, and a meta-analysis. Osteoporos Int.

[11] Cramer JA, Gold DT, Silverman SL, Lewiecki EM (2007). A systematic review of persistence and compliance with bisphosphonates for osteoporosis. Osteoporos Int.

[12] Cortet B, Modi A, Tang J, Fan CP, Sajjan S, Weaver JP (2016). Association between gastrointestinal events and osteoporosis treatment initiation in women diagnosed with osteoporosis in France: a retrospective analysis. BMC Musculoskeletal Disord.

[13] Ross S, Samuels E, Gairy K, Iqbal S, Badamgarav E, Siris E (2011). A meta-analysis of osteoporotic fracture risk with medication nonadherence. Value Health.

[14] Hiligsmann M, Rabenda V, Bruyere O, Reginster JY (2010). The clinical and economic burden of non-adherence with oral bisphosphonates in osteoporotic patients. Health Policy.

[15] Cramer JA, Roy A, Burrell A, Fairchild CJ, Fuldeore MJ, Ollendorf DA, Wong PK (2008). Medication compliance and persistence: terminology and definitions. Value Health.

[16] Arnett FC, Edworthy SM, Bloch DA, McShane DJ, Fries JF, Cooper NS, Healey LA, Kaplan SR, Liang MH, Luthra HS (1988). The American Rheumatism Association 1987 revised criteria for the classification of rheumatoid arthritis. Arthritis Rheum.

[17] Hadji P, Claus V, Ziller V, Intorcia M, Kostev K, Steinle T (2012). GRAND: the German retrospective cohort analysis on compliance and persistence and the associated risk of fractures in osteoporotic women treated with oral bisphosphonates. Osteoporos Int.

[18] Richards JS, Cannon GW, Hayden CL, Amdur RL, Lazaro D, Mikuls TR, Reimold AM, Caplan L, Johnson DS, Schwab P (2012). Adherence with bisphosphonate therapy in US veterans with rheumatoid arthritis. Arthritis Care Res.

[19] Yu SF, Chou CL, Lai HM, Chen YC, Chiu CK, Kuo MC, Su YJ, Chen CJ, Cheng TT (2012). Adherence to anti-osteoporotic regimens in a Southern Taiwanese population treated according to guidelines: a hospital-based study. Int J Rheum Dis.

[20] Cheen MH, Kong MC, Zhang RF, Tee FM, Chandran M (2012). Adherence to osteoporosis medications amongst Singaporean patients. Osteoporos Int.

[21] Soong YK, Tsai KS, Huang HY, Yang RS, Chen JF, Wu PC, Huang KE (2013). Risk of refracture associated with compliance and persistence with bisphosphonate therapy in Taiwan. Osteoporos Int.

[22] Chiu CK, Kuo MC, Yu SF, Su BY, Cheng TT (2013). Adherence to osteoporosis regimens among men and analysis of risk factors of poor compliance: a 2-year analytical review. BMC Musculoskeletal Disord.

[23] Cheng TT, Yu SF, Hsu CY, Chen SH, Su BY, Yang TS (2013). Differences in adherence to osteoporosis regimens: a 2-year analysis of a population treated under specific guidelines. Clin Ther.

[24] Landfeldt E, Strom O, Robbins S, Borgstrom F (2012). Adherence to treatment of primary osteoporosis and its association to fractures--the Swedish Adherence Register Analysis (SARA). Osteoporos Int.

[25] Jacob L, Hadji P, Kostev K (2016). Age-related differences in persistence with bisphosphonates in women with metastatic breast cancer. J Bone Oncol.

[26] Lindqvist E, Saxne T, Geborek P, Eberhardt K (2002). Ten year outcome in a cohort of patients with early rheumatoid arthritis: health status, disease process, and damage. Ann Rheum Dis.

[27] Eberhardt K, Larsson BM, Nived K, Lindqvist E (2007). Work disability in rheumatoid arthritis--development over 15 years and evaluation of predictive factors over time. J Rheumatol.

[28] Prevoo ML, van 't Hof MA, Kuper HH, van Leeuwen MA, van de Putte LB, van Riel PL (1995). Modified disease activity scores that include twenty-eight-joint counts. Development and validation in a prospective longitudinal study of patients with rheumatoid arthritis. Arthritis Rheum.

[29] Cotte FE, Fardellone P, Mercier F, Gaudin AF, Roux C (2010). Adherence to monthly and weekly oral bisphosphonates in women with osteoporosis. Osteoporos Int.

[30] Hansen C, Pedersen BD, Konradsen H, Abrahamsen B (2013). Anti-osteoporotic therapy in Denmark--predictors and demographics of poor refill compliance and poor persistence. Osteoporos Int.

[31] Hoes JN, Bultink IE, Lems WF (2015). Management of osteoporosis in rheumatoid arthritis patients. Expert Opin Pharmacother.

[32] Cooper A, Drake J, Brankin E (2006). Treatment persistence with once-monthly ibandronate and patient support vs. once-weekly alendronate: results from the PERSIST study. Int J Clin Pract.

[33] Ideguchi H, Ohno S, Hattori H, Ishigatsubo Y (2007). Persistence with bisphosphonate therapy including treatment courses with multiple sequential bisphosphonates in the real world. Osteoporos Int.

[34] Silverman SL, Schousboe JT, Gold DT (2011). Oral bisphosphonate compliance and persistence: a matter of choice?. Osteoporos Int.

[35] Kishimoto H, Maehara M (2015). Compliance and persistence with daily, weekly, and monthly bisphosphonates for osteoporosis in Japan: analysis of data from the CISA. Arch Osteoporos.

[36] Iglay K, Cao X, Mavros P, Joshi K, Yu S, Tunceli K (2015). Systematic literature review and meta-analysis of medication adherence with once-weekly versus once-daily therapy. Clin Ther.

